# Spawning induction, development and culturing of the solitary ascidian *Polycarpa mytiligera*, an emerging model for regeneration studies

**DOI:** 10.1186/s12983-020-00365-x

**Published:** 2020-06-11

**Authors:** Tal Gordon, Lachan Roth, Federico Caicci, Lucia Manni, Noa Shenkar

**Affiliations:** 1 George S. Wise Faculty of Life Sciences, School of Zoology, Tel-Aviv University, 6997801 Tel-Aviv, Israel; 2 Department of Biology, University of Padova, 35121 Padova, Italy; 3 The Steinhardt Museum of Natural History, Israel National Center for Biodiversity Studies, Tel-Aviv University, 6997801 Tel-Aviv, Israel

**Keywords:** Tunicates, Model systems, Development, Reproduction, Spawning, *Polycarpa mytiligera*, Red Sea

## Abstract

**Background:**

Ascidians (phylum Chordata, class Ascidiacea) represent the closest living invertebrate relatives of the vertebrates and constitute an important model for studying the evolution of chordate development. The solitary ascidian *Polycarpa mytiligera* exhibits a robust regeneration ability, unique among solitary chordates, thus offering a promising new model for regeneration studies. Understanding its reproductive development and establishing land-based culturing methods is pivotal for utilizing this species for experimental studies. Its reproduction cycle, spawning behavior, and developmental processes were therefore studied in both the field and the lab, and methods were developed for its culture in both open and closed water systems.

**Results:**

Field surveys revealed that *P. mytiligera’s* natural recruitment period starts in summer (June) and ends in winter (December) when seawater temperature decreases. Laboratory experiments revealed that low temperature (21 °C) has a negative effect on its fertilization and development. Although spontaneous spawning events occur only between June and December, we were able to induce spawning under controlled conditions year-round by means of gradual changes in the environmental conditions. Spawning events, followed by larval development and metamorphosis, took place in ascidians maintained in either artificial or natural seawater facilities. *P. mytiligera*’s fast developmental process indicated its resemblance to other oviparous species, with the larvae initiating settlement and metamorphosis at about 12 h post-hatching, and reaching the juvenile stage 3 days later.

**Conclusions:**

*Polycarpa mytiligera* can be induced to spawn in captivity year-round, independent of the natural reproduction season. The significant advantages of *P. mytiligera* as a model system for regenerative studies, combined with the detailed developmental data and culturing methods presented here, will contribute to future research addressing developmental and evolutionary questions, and promote the use of this species as an applicable model system for experimental studies.

## Background

Ascidians are marine invertebrates that constitute the closest relatives to the vertebrate subphylum [1, 2]. They are primarily sessile, with a brief pelagic larval stage that shares the typical chordate tadpole-type features, including a pharynx with gill slits, a notochord, and a hollow nerve cord [3]. Due to their phylogenetic position, inland culturing methods, relatively short life cycle, and simple body plan, ascidians offer an important model for developmental and evolutionary studies [4–7]. Furthermore, their high regenerative abilities make them a key model system for studies seeking to understand the evolution of vertebrate regeneration [8–10].

While many ascidian species are used for cellular and molecular studies, including gene function and regulation during development [7, 10–13], a culturing technique has been established for only a few solitary species, such as *Ciona robusta* (previously called *C. intestinalis* type A [14]), *Ciona intestinalis* and *Halocynthia roretzi* [5, 15–17]. The extensive study of these latter species has led to the development of genetic tools and molecular approaches, including stable transgenic lines [5, 12, 15]. The ability to easily and efficiently breed and maintain a research organism under laboratory conditions is crucial for determining its feasibility to serve as a model system.

The current study focused on the solitary ascidian *Polycarpa mytiligera* (Order: Stolidobranchia). This species is the most common solitary ascidian in the Gulf of Aqaba, Red Sea [18], and an emerging model system for regenerative studies due to its high regenerative abilities [19–21]. These abilities, described also in other *Polycarpa* species, have led researchers in the past to suggest the occurrence of asexual reproduction in this genus [22–24]. However, all the studied *Polycarpa* species to date present a strictly sexual reproduction strategy [25–27].

All ascidians, colonial and solitary, share a similar basic body plan. The adult body consists of a branchial basket, which serves for both the capture of food particles and respiration. The branchial basket opens to the exterior through two anterior siphons and is connected to the posterior digestive track [23]. The neural complex is located between the siphons and comprises a single cerebral ganglion with an associated glandular organ called the neural gland [28, 29]. The ascidian body plan can develop through two very different pathways, sexual or asexual. This diversity of life-history strategies provides a unique opportunity for comparative developmental studies.

Ascidians are hermaphrodites, possessing both male and female organs. Solitary ascidians, such as *P. mytiligera*, release their gametes into the seawater by spawning, and the gametes are then fertilized externally [30, 31]. Spawning events demonstrate species specificity and are affected by different environmental cues, such as light, food availability, and regional variations in water temperature [32–35]. Following fertilization, swimming tadpole-like larvae develop. The larvae have an important role in dispersal and settlement. In free-spawning species they can swim for between 12 h to several days [36–38].

Ascidian larval recruitment varies among seasons, influenced by the ecological conditions and the species-specific interactions with the environment [39–41]. Following settlement, the larvae undergo metamorphosis, a complex process that involves various morphogenetic and physiological changes in the animal [42, 43]. Upon onset of metamorphosis, the larva attaches to the substrate via its anterior adhesive papillae [42], followed by tail resorption, which involves the loss of the notochord, hollow dorsal nerve cord, and the post-anal tail. A rotation of the body axis then begins, followed by the opening of the siphons, beating of the heart, and the appearance of blood cells, indicating that the animal has entered the juvenile stage [44]. At this stage, the growth of adult organs such as the gonads, and the continuing development of the branchial basket and digestive system, occur [44, 45].

Here we have developed a protocol for the spawning and culturing of *P. mytiligera* in captivity, based on field observations and controlled laboratory experiments. Using light and confocal microscopy, histology, and immunocytochemistry methods we provide the first detailed description of its early life stages, from a swimming larva to a metamorphosed juvenile. In addition to contributing to knowledge of the development, larval morphology, and metamorphosis in ascidians, this work presents a fundamental step toward establishing *P. mytiligera* as a new and valuable model system for developmental and regenerative studies.

## Methods

### Field surveys

Quantitative surveys were used to document *P. mytiligera* recruitment patterns along the north shore of the Gulf of Aqaba, Red Sea (Eilat, Israel).

Belt transects [46, 47] were deployed from a random start point, at 15 m and 5 m depth, along the Inter-University Institute (IUI) south shore (29.501435″N, 34.917521″E). Following selection of the start point, four belt transects (15 m × 1 m), were permanently marked at 10 m intervals. The transects were monitored by SCUBA surveys on a monthly base for an 18-month period, from April 2015 to September 2016. During the surveys the number of *P. mytiligera* individuals was noted and their size was measured.

### Animal collection for spawning experiments

**Fig. 1:**
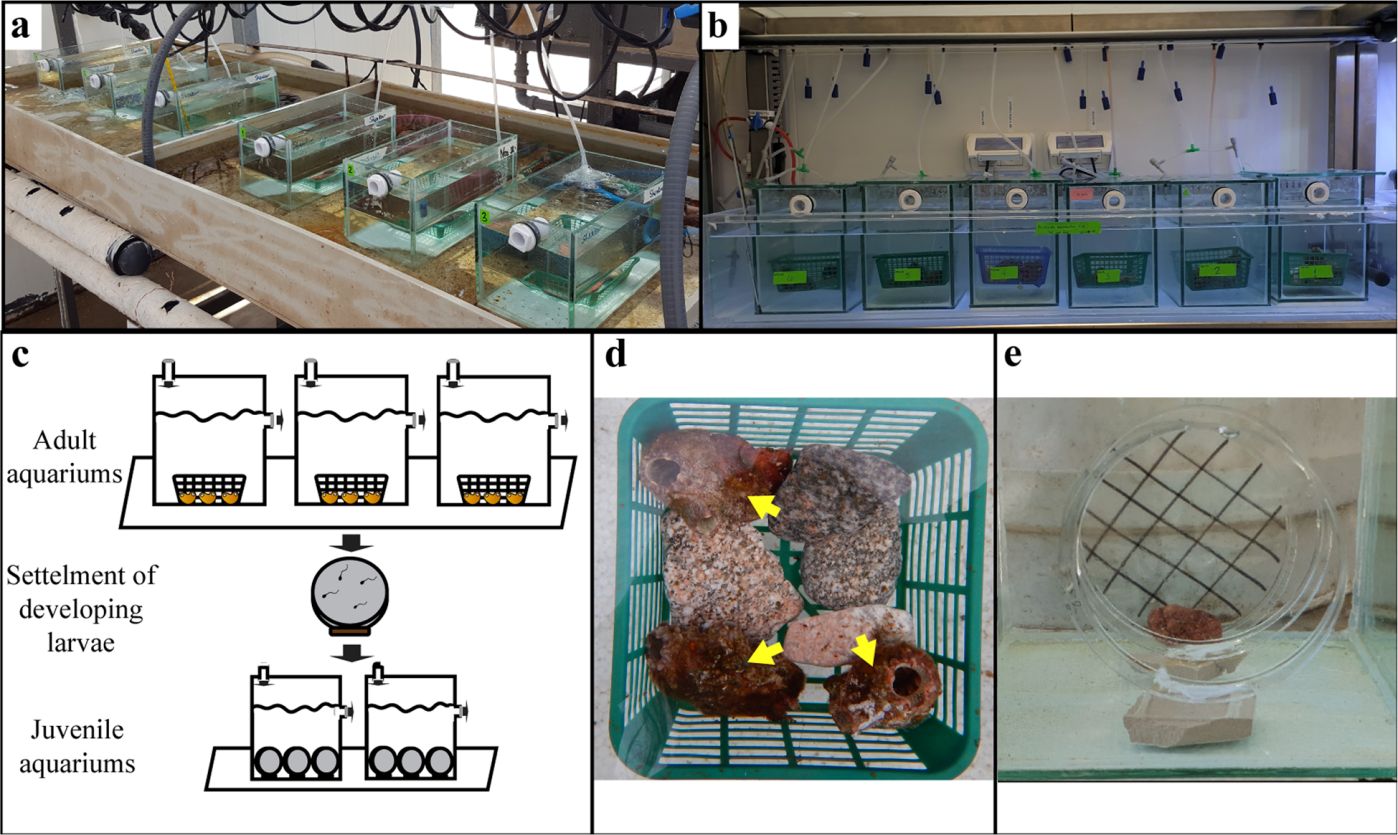
Experimental design. **a** The open water system (System A). **b** The closed water system. **c** Illustration of the experimental design. **d** Adult ascidians (yellow arrows) in the aquarium system. **e** Petri dishes with settled larvae held vertical in the juvenile aquarium

*P. mytiligera* spawning and reproduction were studied in two aquarium systems: an open water system connected to the Red Sea at the Inter-University Institute (IUI) in Eilat, Israel (Fig. 1a); and a closed water system located at Tel Aviv University, Tel Aviv (Fig. 1b). Adult animals were randomly collected by SCUBA diving from the north shore of the Gulf of Aqaba, Red Sea (29.547440″N, 34.954084″E). The collected animals were transferred to a water table with running seawater (~ 80 GPH) at the IUI, and kept under ambient conditions for 24 h of acclimation prior to beginning the experiments. For experiments in the closed water system at Tel Aviv University (Tel Aviv, Israel), live animals were transferred in a sealed box filled with seawater. Temperature was maintained using a cooler box. Animals were kept for 24 h of acclimation at a Tel Aviv aquarium facility prior to beginning the experiment. All animals were of similar size range (5 ± 1 cm in length measured from the tip of the oral siphon to the base of the animal).

### Open water system

We set up an outdoor open water system at the IUI (system A) and an indoor open water system at the National Center for Mariculture in Eilat, Israel (system B). Each system comprised nine aquaria (30 × 23 × 30 cm^3^) supplied with fresh seawater.

The outdoor system (system A) maintained a natural seasonal day-night routine (Additional file 1). A shading net was used to reduce light intensity in order to resemble that at the collection site. This system was used for experiments simulating natural environmental conditions (i.e water temperature and day-night cycle).

The indoor system (system B) enabled control of the artificial environmental conditions (light intensity and day-night routine) by means of a set of neon lamps (18 W/865, Osram) located above the system.

In both systems seawater entering and exiting the aquariums was filtered through a 100 μm mesh, preventing large particles from entering or exiting the system. Air was supplied via a stone diffuser in all aquaria. Water temperature was set using heaters or chillers according to the desired treatment in both systems.

#### Experimental design

Due to *P. mytiligera’s* gonadal structure, direct collection of sperm and eggs from dissected animals is a complex process with a low fertilization success rate (T. Gordon, unpublished data). Gametes were therefore collected via the natural spawning process. The ascidians’ tunics were carefully cleansed of epibionts and the animals were placed in the experimental aquarium system. Each aquarium held three individuals anchored to the bottom of the aquarium by small rocks (Fig. 1c, d). We chose to use only three animals per aquarium, which is the minimum required for natural external fertilization, in order to minimize impact on the natural population. Each aquarium is referred to as one repetition in the different experiments. The animals were observed daily to monitor their viability. Feeding was provided by the natural small food particles entering the system via the water current. To evaluate spawning success, 25% of the water volume in each aquarium was filtered, using a 65 μm net, transferred to a sterile Petri dish, and scanned for reproduction products. Eggs and the post-hatching stages including swimming larvae, settled larva, and metamorphosed juveniles were collected from the water column and aquarium walls. Reproduction products in the Petri dishes were carefully counted under a microscope (Nikon SMZ18) and transferred for settlement (section 2.5). No discrimination was made between fertilized and unfertilized eggs at this stage, and a successful spawning event was determined according to the presence of live post-hatching stages in the aquaria.

#### Temperature effect on spawning during the natural reproduction season

The effect of seasonal variation in water temperature on spawning success, larval hatching, and metamorphosis was examined during the natural reproduction season (August 2016), under different temperature treatments. Aquarium system A was divided into three treatments: high temperature (30 °C), cold temperature (21 °C), and control with ambient temperature (27 °C). The selected high and low temperatures were based on the maximum and minimum temperature in the Gulf of Aqaba [48]. Three aquaria (total of nine individuals) were used for each of the treatments and the aquaria were scanned for reproduction products daily for 6 days, a time period in which the main spawning event took place (T. Gordon, personal observations).

#### Spawning induction - effect of seasonal variation and heat shock

To examine the effect of seasonal variation on spawning success in captivity, spawning was observed under natural conditions versus experimental conditions throughout the year in the outdoor system (system A). The environmental conditions in system A were set according to the natural conditions at the collection site (Additional file 1). As increased temperature is often used to induce spawning when culturing marine invertebrates [49–51], the effect of a thermal shock of 30 °C, two degrees above the maximum temperature in their natural habitat [48], was examined as a potential cue for inducing spawning in *P. mytiligera*. System A was divided into two treatments: a) heat treatment, in which the animals experienced a thermal shock; and b) control treatment, maintaining the natural conditions according to the current season. Sample size and environmental conditions in system A throughout the different seasons are provided in Additional file 1.

The animals spent no longer than three full day-night cycles at the elevated temperature in order to avoid a cumulative stress reaction [52, 53]. All aquaria were scanned for reproduction products daily for 6 days following the heat treatment.

#### Spawning induction outside the natural reproduction season

To enable year-round spawning, we applied a gradual change from winter (21–22 °C)/ spring (22–23 °C) conditions to summer conditions (27–28 °C) in aquarium system B. In February 2018 and April 2019, indoor aquarium system B was divided into two treatments: a) control - three aquaria maintaining the natural winter/ spring conditions; and b) heat treatment – three aquaria experiencing a gradual shift in water temperature and day length. A total of nine individuals were used per treatment. Starting conditions for both treatments were according to the natural season conditions (Additional file 1). The control treatment maintained these starting conditions for the entire experimental period, while the treatment aquaria received an increase of 1 °C every 2 days and elongation of day length by 15 min every 2 days, until reaching ambient summer conditions (27–28 °C, see Additional file 1).

### Closed water system

A closed aquarium seawater system was set up in the aquarium facility at Tel Aviv University in order to study *P. mytiligera* reproduction success. The system comprised six aquaria (15 × 28 × 20 cm^3^) filled with artificial seawater (ASW). Air was supplied via a stone diffuser in all aquaria and the temperature was set using electric water heaters or chillers. A set of lamps (LED Submersible lamp, RGB 1350 mm, AQUADECOR) located above the aquarium system was used to set a day-night routine.

ASW was kept in a 270 L reservoir tank, connected to a second, 35 L tank, in which the ASW was prepared and filtered. The ASW tank was connected to a pump system mixing deionized water with salt crystals (Red Sea Salt, Red Sea), in order to achieve a final ASW salt concentration of 40 g/L presenting a similar chemical composition to that of the Red Sea natural seawater. The ASW was pre-filtered using a protein skimmer and UV light before entering the aquarium system. Water temperature in the system was maintained by a heater at 27 °C.

#### Reproduction study in the closed water system

Acclimation, cleaning, and viability examination of the adult animals were performed as described previously for the open water system. Animals (*n* = 12) were divided into six aquaria. Each aquarium held two individuals anchored to the bottom by small rocks (Fig. 1c, d). We chose to use only two animals per aquarium based on preliminary experimental results showing a low survival of adults at a higher density in a closed system (T. Gordon, unpublished results). Animals were fed daily with a live unicellular algal mix (*Hymenomonas sp*., *Isochrysis sp*. and *Cheaetoceros sp*. obtained from the National Center for Mariculture in Eilat, Israel) of 2 × 10^7^ cells/ml. Evaluation of spawning success was carried out similarly to that in the open water system. A quarter of the volume for each aquarium was replaced every other day with fresh ASW to prevent the accumulation of ammonia waste and to examine the water for reproduction products. To evaluate spawning success, a water sample from each aquarium was filtered, using a 65 μm net, transferred to a sterile Petri dish, and scanned for reproduction products. These products were carefully counted under a microscope (Nikon SMZ18) and transferred for settlement (section 2.5). Water temperature (27–28 °C) and day length were set to resemble the natural summer conditions in the Red Sea (Additional file 1). The aquaria were scanned for reproduction products every 2 days for a five-week period.

### Juvenile settlement and development in the open and closed systems

Following a spawning event, the reproduction products were counted, and the live products were carefully transferred using a glass pipette to new Petri dishes with filtered seawater (in the open water system) or to ASW (in the closed system). Up to 25 live products were transferred to each Petri dish and positioned horizontally for 24 h to allow settlement (Fig. 1c). Dead products were identified according to the deformation of body form and were omitted from the live product count. Following settlement, the Petri dishes were deployed vertically (by anchoring them with a small stone) in the aquarium (15 × 15 × 15 *cm*^3^) (Fig. 1c, e). The natural gravity of vertical orientation kept the juveniles free from the accumulation of sediment and debris. In the open system the aquaria received a constant supply of filtered seawater (100 μm filter) to create a constant water turnover (Fig. 1a). In the closed water system, the aquaria were filled with ASW, which was changed daily. The water in the aquaria was constantly aerated through a stone diffuser (Fig. 1b). Water temperature was maintained at 25 ± 1 °C, and the aquaria and Petri dishes with settled juveniles were kept clean by means of daily water exchange and the careful removal of particles from the Petri dishes using a paintbrush.

The survival and developmental process of the transferred reproduction products were followed and documented daily.

### Histology

In order to describe the *P. mytiligera* gonadal structure, three random adults of similar size range (5 ± 1 cm, from the tip of the oral siphon to the base of the animal) were collected during the summer (August 2018). The animals were fixed in 4% formaldehyde in seawater after relaxation with menthol, separated from their tunic, and dehydrated with ethanol at increasing concentrations prior to embedding in paraffin (Paraplast Plus, Lecia). Sections (7 μm) were mounted on glass slides, deparaffinized, and stained with standard hematoxylin and eosin solutions. For a detailed description of *P. mytilgera* early life stages morphology, selected larvae and juveniles (1 week old) were fixed in 1.5% glutaraldehyde buffered with 0.2 M sodium cacodylate, pH 7.4, plus 1.6% NaCl. After washing in buffer and post-fixation in 1% OsO_4_ in 0.2 M cacodylate buffer, the specimens were dehydrated and embedded in epoxy resin (Sigma-Aldrich). Sections (1 μm) were counterstained with toluidine blue for morphological analysis.

### Immunolabeling of *P. mytiligera* early life stages

For immunolabeling, *P. mytiligera* larvae (*n* = 10) and juveniles (1 week old, *n* = 10) were fixed in 4% paraformaldehyde in phosphate buffered saline (PBS) overnight at 4 °C, and washed three times (5 min each) in PBS followed by a 10 min wash in TritonX-100 at room temperature (RT). Samples were washed twice (10 min each) in PBT (PBS + 0.2% Tween20) and blocked in PBT + 3% BSA for 3 h at RT. Antibodies were then added directly to the blocking solution overnight at 4 °C. To visualize nerves and cilia, we used a mouse monoclonal anti-acetylated tubulin antibody (Sigma T7451) diluted 1:1000 in blocking solution [54–57]. Samples were then washed twice for 10 min each in PBT. Secondary antibodies (ThermoFisher, Alexa Fluor goat anti-mouse IgG 488 A-11001) and phalloidin (ThermoFisher, Alexa Fluor A-2228) were added at 1:500 dilution to the blocking solution for 3 h at RT. Samples were then washed in PBS three times for 10 min each. Samples were stained with Hoechst (ThermoFisher 33,342) (1 μg/mL in PBS), and mounted in VECTASHIELD (Vector Laboratories RK-93952-28) using coverslips. The negative control samples were processed without incubation in primary antibodies. No detectable fluorescence was exhibited by the specimens in the control experiments. Samples were imaged on a Leica SP5 with 10X, 20X and 40X objectives.

### Statistical analysis

Statistical analysis was performed using R statistics (R Core Team, 2014) with RStudio (RStudio Team, 2012). Significant values for all tests were regarded as *P value* < 0.05.

Permutation analysis of variance (ANOVA) was applied to test for differences in *P. mytiligera* population density and individual size along the different seasons. Variation in the proportion of newly recruited individuals in the field was determined by permutation ANOVA following arcsine square root transformation.

To determine whether the amount and type of reproduction products produced during the experiment period are affected by temperature and day length, we used a permutation ANOVA, with temperature, day, and product type, as well as their interaction, as factors.

All permutational ANOVA tests were performed using the RVAideMemoire package and calculated *P*-values using 9999 random permutations [58] followed by pair-wise permutations tests for determining within-factor differences. Pairwise comparison values were corrected using the Benjamini & Hochberg (FDR) false-discovery rate control, setting an error rate of *p* = 0.05 [59].

## Results

### Field surveys

**Fig. 2:**
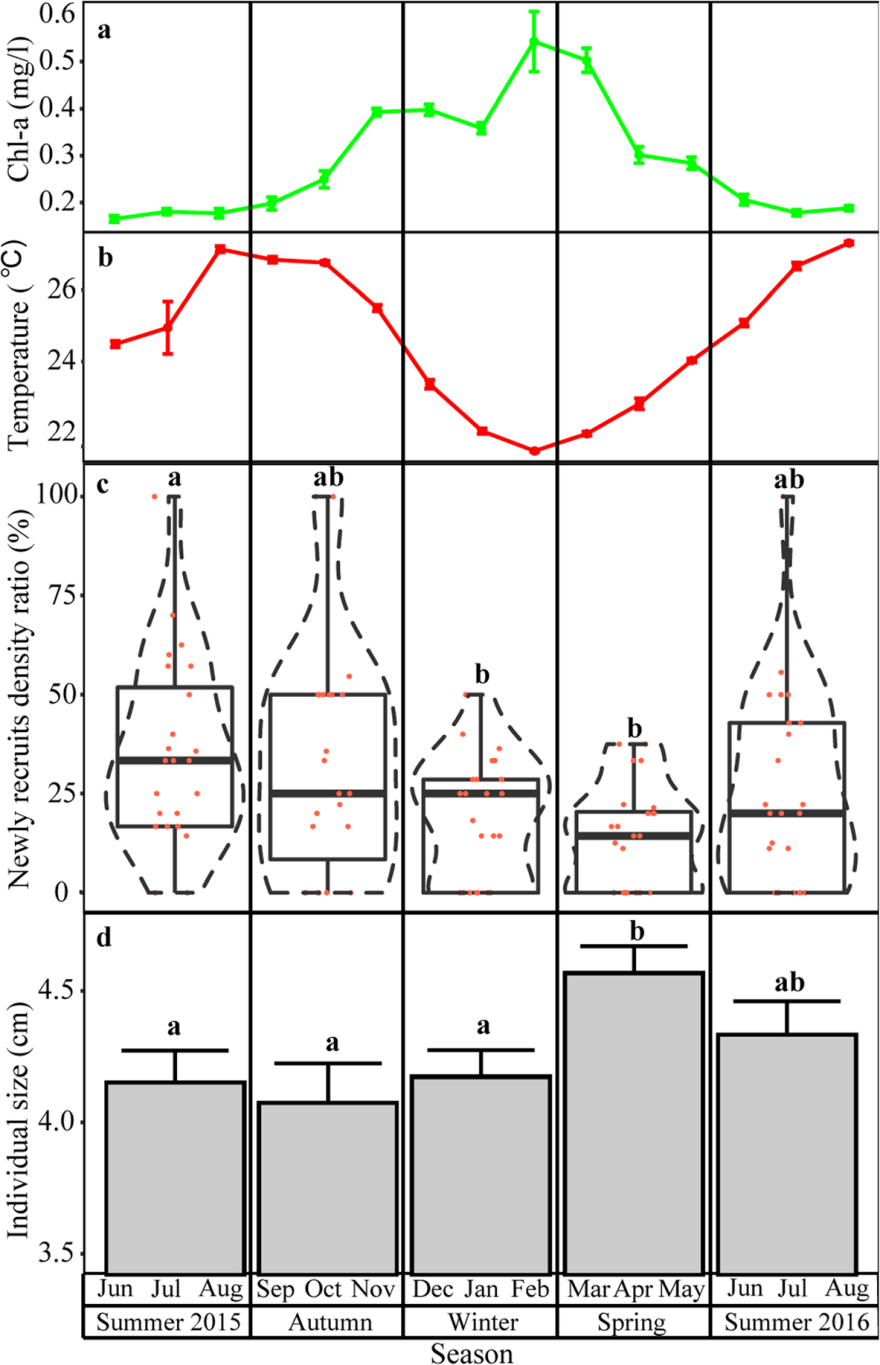
Seasonal variation in *P. mytiligera* population structure in relation to chlorophyll- a concentration and sea temperature. **a** Variation in mean (±SE) of chlorophyll- a concentration and **b** sea temperature in the Red Sea. **c** Violin plots display the proportion (%) of *P. mytiligera* new recruit density in relation to total population density. The plot diameter reflects the probability density of new recruits for each season. Jittered points overlaid on boxplots represent individual samples. The bottom and top of the boxes represent the first and third quartiles, the central band represents the median, and whiskers represent 1.5-fold the interquartile range. **d***P. mytiligera* seasonal average size in cm (±SE). Data averages were calculated from a unit of 15 m^2^ belt transect (*n* = 24 belt transects per season). Different letters above bars indicate significant differences (permutational ANOVA, *p* < 0.05)

Field surveys were conducted in order to document the effect of seasonal variation on *P. mytiligera’*s natural population recruitment pattern. The seasonal changes in chlorophyll- a concentration and seawater temperature during the survey period are illustrated in Fig. 2a-b (data summarized from the National Monitoring Program reports [48] which provide environmental data from 2004). A significantly higher number of newly-recruited individuals (≤ 3 cm) was found during the summer of 2015 in comparison to the winter and spring (permutational ANOVA, *p* < 0.05, *n* = 24 transects per season) (Fig. 2c). Size distribution results revealed the presence of significantly larger individuals (permutational ANOVA, *p* < 0.05) during the spring (*n* = 158) in comparison to autumn (*n* = 104), summer (*n* = 168), and winter (*n* = 157) (Fig. 2c, d). A comparison of number of *P. mytiligera* individuals among seasons revealed significantly higher numbers in the summer, winter, and spring than in the autumn (permutational ANOVA, *p* < 0.05, *n* = 24 transects per season) (Additional file 2).

### Spawning success in the open-water system

*P. mytiligera*’s ability to spawn under laboratory conditions was studied in order to develop a protocol for the maintenance and culturing of this species in captivity. Ascidians maintained in captivity were shown to spawn in both the outdoor and indoor systems. Fertilized eggs were found in the aquaria only at sunset. Larvae, in various stages of metamorphosis, were found the following morning. The swimming larvae that were transferred to Petri dishes initiated metamorphosis and successfully developed into the juvenile stage.

#### Temperature effect on spawning during the natural reproduction season

**Fig. 3:**
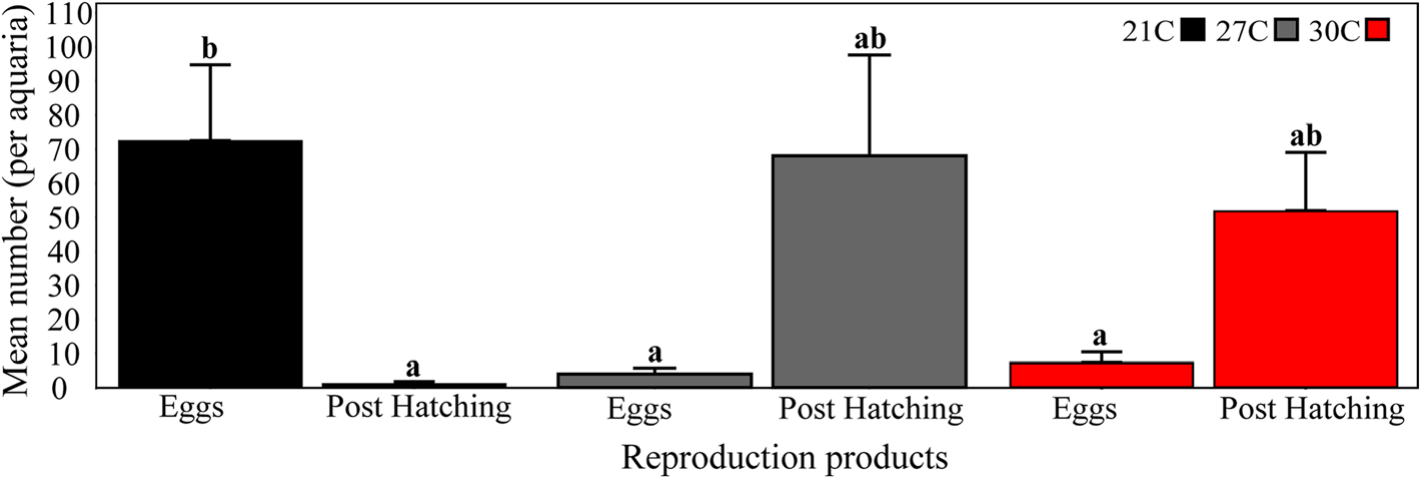
*P. mytiligera* spawning productivity in an open water system under different temperature treatments. Mean number (±SE) of *P. mytiligera* reproduction products (eggs and post hatching stages) under the three temperature treatments: cold (21 °C), heat (30 °C), and control (27 °C) throughout the six days of experiment (*n* = 3 aquaria/ treatment). Different letters above bars indicate significant differences between reproduction products of the same treatment (permutational ANOVA, *p* < 0.02)

Different temperature treatments were used to examine the effect of temperature on spawning success and development during *P. mytiligera*’s natural reproduction season. Spawning events were documented in all treatments (*n* = 3 aquaria per treatment): low (21 °C), control (27 °C), and high temperature (30 °C). The average number of eggs in the low temperature treatment was significantly higher (permutational ANOVA, *p* < 0.05) compared to the number of post-hatching stages (larva and juvenile) of the same treatment; and also higher than the number of eggs in the control and high-temperature treatments (Fig. 3).

#### Spawning induction- effect of seasonal variation and heat shock

To examine the effect of seasonal variation on spawning success in captivity, spawning events were monitored throughout the year. Spawning under both natural and thermal shock conditions (Additional file 1) were documented in the summer and autumn but not in the spring and winter. During the summer and autumn seasons about 80–100 reproduction products per aquarium (eggs, larvae, and juveniles) were observed in both the control and high temperature treatments.

#### Induced spawning outside the natural reproduction season

To enable year-round spawning, we studied the effect of gradual environmental change on *P. mytiligera*’s spawning success. A gradual shift in water temperature and day length from winter/spring conditions to summer conditions led to spawning events in all of the treatment aquaria (*n* = 3 aquaria). No spawning events were seen in the three control aquaria maintaining the natural winter/spring conditions. Reproduction products (ca. 40–50 per aquarium) were observed in all the treatment aquaria after 25–35 days under controlled summer conditions.

### Spawning success in the closed-water system

**Fig. 4:**
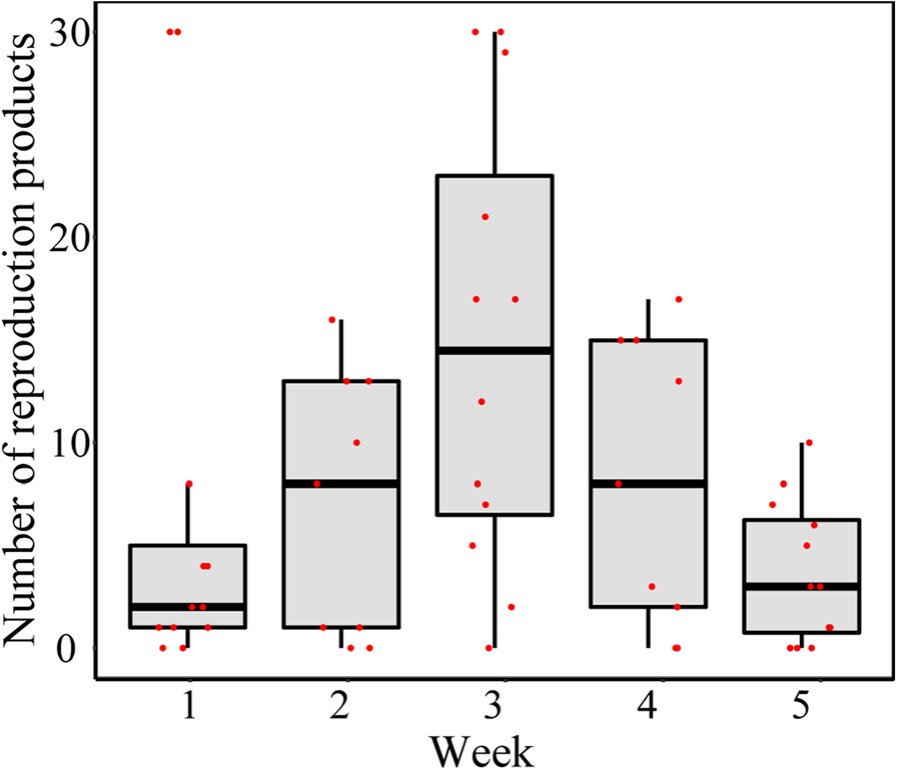
Weekly variation in *P. mytiligera* spawning productivity in the closed water system. Weekly variation in *P. mytiligera* reproduction products throughout the five-week period (*n* = 3 aquaria). Jittered points overlaid on boxplots represent individual samples. The bottom and top of the boxes represent the first and third quartiles, the central band represents the median, and whiskers represent 1.5-fold the interquartile range

To evaluate *P. mytiligera*’s adaptation to the ASW-based facility, its survivability and reproduction success were studied. Successful spawning was displayed by the ascidians maintained in the ASW-based facility at Tel-Aviv University (*n* = 6 aquaria). During the 5 weeks of the experiment several spawning events occurred in the closed system, releasing gametes into the water column. Eggs were successfully fertilized and developed into swimming larvae, which then metamorphosed and developed into juveniles. Post-hatching stages were found during all 5 weeks of the experiment (Fig. 4). The mean survival percentage ± SE of settled post-hatching stages for a one-week period was 45.8% ± 8% (*n* = 27 settlement plates, *n* = 272 settled individuals).

### Gonad histological structure

Histological sections were used to describe the *P. mytiligera* gonadal structure. *P. mytiligera* is characterized by multiple short hermaphrodite gonads (polycarps) embedded in the body wall [60]. Histological cross-sections of the body wall revealed that each polycarp comprises a testis composed of several follicles, and an ovary with several oocytes. The oocytes are on the opposite side of the polycarp with respect to the testis and are grouped around a ciliated oviduct (Additional file 3).

### *P. mytiligera* developmental process

**Fig. 5:**
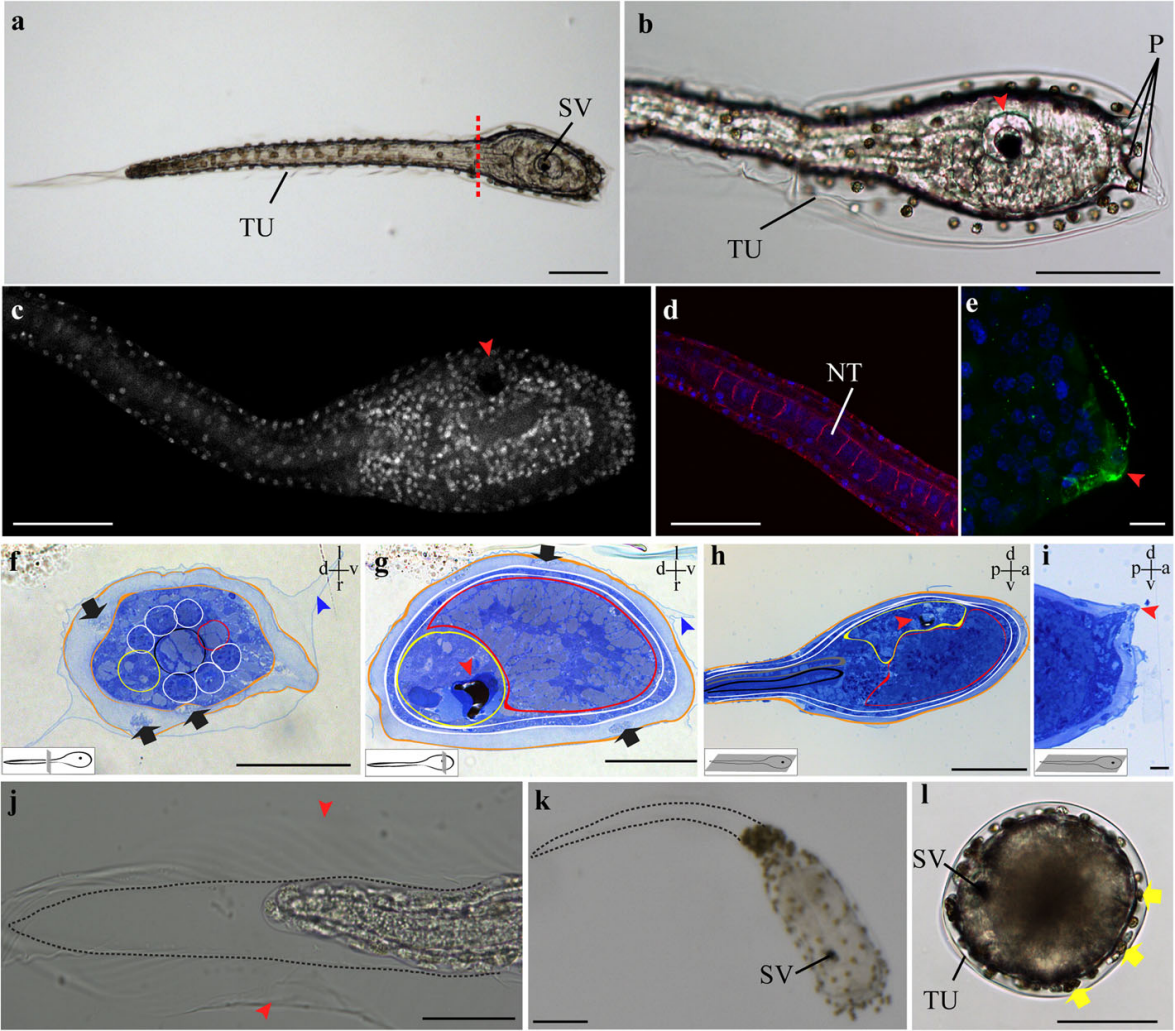
Larval development and settlement in *P. mytiligera*. **a-b**, **j-l** Light micrographs of live animals. **c**-**e** Confocal images. Larvae are labeled with three markers: phalloidin (red), anti-acetylated tubulin (green), and Hochest (blue/gray). **f**-**i** Histological sections, Toluidine blue. **f**, **g** cross-sections, **h**, **i** sagittal sections. Section level and axis are shown in a scheme in the lower left corner and upper right corner of each histological image. All panels are anterior to the right unless otherwise indicated. **a-i** swimming larva stage; **j**-**l** metamorphosis. **a** Dashed line marks the boundary between the anterior trunk and posterior tail. **b-c** Larval trunk. Red arrowheads indicate the sensory vesicle. **d** Detail of larval tail. Note the notochord (NT) cells. **e** Detail of an anterior sensory papilla (red arrowhead). **f** Cross-section of larval tail showing notochord (black), endodermal strand (red), muscle strands (white), nerve cord (yellow), inner tunic layer (orange). Black arrows indicate tunic cells; blue arrowhead indicates the outer layer of tunic. **g** Cross-section of larval trunk showing pharynx (red), epidermis (white), visceral ganglion (yellow), and inner tunic layer (orange). Red arrowhead indicates the sensory vesicle, black arrows indicate tunic cells, blue arrowhead indicates the outer tunic layer. **h** Sagittal section of the larval anterior body showing notochord (black), nerve cord (gray), pharynx (red), visceral ganglion (yellow), epidermis (white), inner tunic layer (orange). Red arrows indicate the sensory vesicle. **i** Sagittal section of the larvae anterior sensory organs indicated by red arrows. **j** Detail of the resorbing tail in a metamorphosing individual. Red arrowheads indicate the outer layer of tunic, dashed line marks the original tail boundaries. **k** Settled larvae. Dashed line marks the original tail boundaries. **l** Top view of a larva immediately following adhesion. Yellow arrows indicate tunic cells. TU: tunic. Scale bar in A-D, H, K, L: 100 μm; in F, G, J: 50 μm; in E, I: 10 μm

**Fig. 6:**
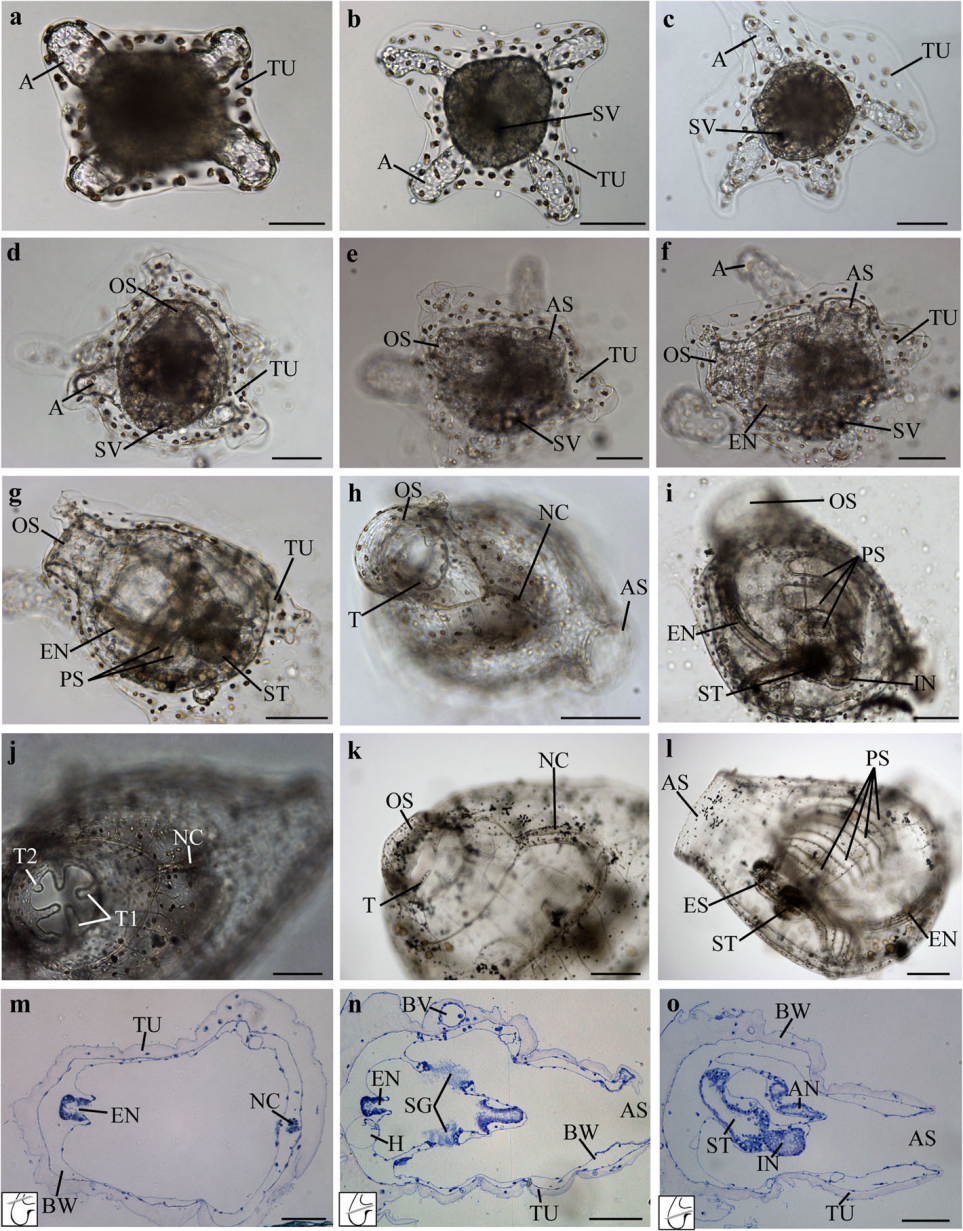
Metamorphosis process and juvenile development in *P. mytiligera*. **a**-**f** Metamorphosis process and early juvenile development, light micrographs of live animals. **a-e** Body axis rotation stage. **a** 3 h, **b** 11 h, **c** 24 h, **d** 33 h, and **e** 45 h post-settlement. **f** Juvenile stage, 50 h post-settlement. **g-o** Juvenile development. **g-l** Light micrographs of live animals. Anterior at top. **m-o** Histological cross-section, 7 days post-settlement. Scheme of the section level is shown in the lower left corner of each image. Ventral at left. **g-h** 3 days post-settlement: **g** 58 h and **h** 70 h post-settlement. **i** 5 days post-settlement. **j** 13 days post-settlement. **k-l** 58 days post-settlement. Ampulla (A), atrial siphon (AS), anus (AN), tunic blood vessel (BV), body wall (BW), digestive system (DS), endostyle (EN), esophagus (ES), heart (H), neural complex (NC), oral siphon (OS), pigmented sensory organ (SO), protostigmata (PS), stomach (ST), tunic (TU) and tentacles (T1 first order, T2 second order). Scale bar in A-K: 100 μm; in L: 200 μm; in M-O: 50 μm

The development process from the swimming larval stage to the juvenile stage was closely monitored and photographed at 25C° for a period of 60 days (Figs. 5 and 6).

#### Embryonic and larval development

*P. mytiligera* releases its gametes into the water, where fertilization occurs. The eggs are brown in color and measure approximately 200 μm in diameter. The complete embryogenesis from fertilized egg to a swimming larva lasts 11 h at 25 °C (Additional file 4). The larva, 1 mm long, is composed of an anterior trunk (258 ± 53 μm, *n* = 15) and a posterior tail (Fig. 5a-c). The trunk comprises the rudiment of the pharynx and the dorsal nervous system (Fig. 5b, g, h). The nervous system comprises a visceral ganglion, a sensory vesicle with a single pigmented sensory organ, and a hollow dorsal nerve cord extending posteriorly into the tail (Fig. 5f-i, Additional file 5). In the anterior trunk, three apical sensory adhesive papillae are discernible (Fig. 5b, e, i). The tail has a central notochord flanked by three paired bands of muscle cells (Fig. 5d, f). The nerve cord lies dorsal to the notochord (Figs. 5f-h). The larva’s epidermis is covered by a double layer of tunic (Fig. 5b, f-g): the thick inner layer contains large brown/yellow cells; while the outer layer extends to form fins along the dorsal-ventral axis at the tail (Fig. 5f, g, j, l).


**Additional file 4: Movie 1.** Time lapse of *P. mytiligera* embryogenesis process.


#### Metamorphosis

Following a swimming stage of about 12 h, the larva adheres to the substrate with its papillae. The metamorphosis process begins with the absorption of the tail, the notochord, and the hollow dorsal nerve cord (Fig. 5j-l, Additional file 6). During this developmental phase, the trunk length increases from 125 μm in the swimming larva to 300 μm in a settled individual. The animal then loses all larval-specific structures of the body plan except the sensory vesicle and pharynx.


**Additional file 6: Movie 2.** Time lapse of *P. mytiligera* larvae tail absorption process*.*


Following absorption of the tail (Fig. 5j, k), rotation of the body axis begins, and the animal reorganizes into the adult body plan (Fig. 6). The oral and atrial siphon rudiments appear in the first 48 h post-settlement (Fig. 6d, e). Both siphons are closed at this stage but are able to contract. Blood ampullae, joined through a blood vessel, elongate into the basal tunic that anchors the animal to the substrate (Fig. 6). On Day 3, the animal opens its siphons and enters the juvenile stage (Fig. 6f).

#### Juvenile development

The juvenile presents the typical adult body plan (Fig. 6, Additional files 7, 8). Three days post- settlement the first two protostigmata are visible and active (Fig. 6g) and the heart begins to beat. Moving cells are visible in the blood vessels. Oral tentacles of the first order are recognizable at the base of the oral siphon (Fig. 6h). On Day 5, three protostigmata are visible and the endostyle can be distinguished in the ventral pharynx (Fig. 6i). On Day 7, the digestive system is differentiated, displaying the esophagus, stomach, and intestines (Fig. 6l, n, o, Additional file 8). The neural complex, now discernible, incorporates the cerebral ganglion and a neural gland with a single ciliated aperture in the pharynx (Fig. 6j, k, m, Additional file 8). On day 13, tentacles of the first and second order are visible in the oral siphon (Fig. 6j).


**Additional file 7: Movie 3.***P. mytiligera* 13-day old juvenile showing the different body structure and organs. Scale bar: 100μm*.*


## Discussion

A limited supply of animals is a major obstacle in the use of new model animals for experimental studies. In many cases, and especially in developmental studies, researchers depend on their model animal’s natural reproduction period for the collection of mature adult specimens from the field to spawn in the lab. This limitation has forced researchers to focus on only a few well-established model animals. Our current study is thus important in presenting a culturing technique for a promising new model animal: the solitary ascidian *Polycarpa mytiligera*.

### *P. mytiligera*’s natural reproductive cycle exhibits seasonal fluctuation

The seasonal variation in population density and in body size observed in *P. mytiligera* population in the field, based on 18 months of surveys, suggests that this species’ recruitment period starts in the summer and ends with the onset of winter.

Water temperature has been found to have an important influence on the growth and reproduction cycles of ascidians [30, 34, 61] as well as on their recruitment [62–64]. Increased water temperature in the summer induces spawning in *P. mytiligera,* resulting in a higher density of new recruits during this season in comparison to the winter and spring.

In the autumn the average size of individuals was at its lowest, due to the settlement of new recruits and the mortality of adults (the latter may be related to the decline in food availability and the drop in water temperature) [65, 66]. A relationship between mortality and low temperature was also found in the colonial ascidians *Botryllus eilatensis* from the Red Sea [67] and *Botryllus schlosseri* and *Botrylloides violaceus* in British Colombia [68], where low temperature had a negative effect on colony size and survival. Larger individuals were found in the spring, following the vertical mixing event [65, 66], as food availability was then maximal.

The results from the open water system spawning experiment support our hypothesis of the summer and autumn as *P. mytiligera*’s natural reproduction seasons. During the summer and autumn gametes and larvae were found in both the control and the high temperature treatment, indicating that reproduction can be easily obtained with no external induction required during this time of the year. No reproduction products were found during the winter and the following spring in both the high temperature and the control treatments. These findings imply that *P. mytiligera* gonads are not mature during winter and were therefore unable to undergo spawning despite the thermal shock treatment. Previous studies have shown that ascidian spawning events are affected by both food availability and water temperature [32–35]. Since chlorophyll concentration is comparatively high in the Gulf waters during winter and spring, *P. mytiligera* reproduction was probably inhibited by other parameters, such as low water temperature and shorter day length, which were not yet optimal for spawning. It is therefore possible that during winter and spring the mature animals need to acquire the energy necessary for gonad development towards the summer season, when water temperature rises to a level that allows their reproduction. A similar reproduction cycle was found in other ascidian species from the Red Sea. The colonial ascidian *Botryllus eilatensis* and the solitary ascidian *Halocynthia spinosa*, both native to the Red Sea, display a seasonal reproduction period that correlates with the summer [39, 69].

The prominent effect of temperature on *P. mytiligera* spawning was demonstrated in our low temperature experiment. While cold temperature did not prevent spawning and the release of a high number of eggs during the natural reproduction period, it did affect their further development into larvae and juveniles. The negative effect of low temperature on fertilization and development in *P. mytiligera* further supports the inability of this species to reproduce during the winter. Similar results were achieved by Batty et al. [70], showing that low temperature had a negative effect on the development and survival of the ascidian larva *Dendrodoa grossularia*. Elevating water temperature above the natural summer conditions was shown here to have no significant effect on spawning, fertilization, or development, and should therefore be excluded from the culturing protocol of *P. mytiligera*.

### Artificial spawning can be obtained year-round, independent of the natural reproductive season

The current study has demonstrated that *P. mytiligera* adults can be induced to spawn at any given time throughout the year, enabling a constant supply of reproduction products for long-term experiments. A gradual experimental shift in water temperature and day length led to spawning events taking place outside of this species’ natural reproduction period. During this experiment, water temperature and day length were artificially and gradually altered from winter/spring conditions to summer conditions, resulting in spawning success for the experimental animals, but not for the control, which had maintained the natural winter/spring conditions. Furthermore, the spawning events were followed by successful larval development and metamorphosis into settled juveniles, indicating the high quality of the released gametes. The current protocol utilizes the animal’s natural spawning behavior in order to collect gametes, as in-vitro fertilization results in low fertilization success due to the gonads’ small size and difficulty in discerning and dissecting the genital ducts. Although in-vitro fertilization is better controlled, offering an advantage for screening for developmental mutations and transgenic lines, the collection of gametes by natural spawning enables the adults to be kept for repeated spawning and for further genetic studies [5, 15].

### *P. mytiligera* as a promising model for comparative developmental studies

The introduction of *P. mytiligera* adults into artificial conditions in order to induce spawning was highly successful, with 100% survival in all treatments. Adult animals exhibited repeated spawning events during the 5 weeks of the experiment. Furthermore, gamete release was followed by successful fertilization and development into larvae, which metamorphosed into settled juveniles that continued to develop under the artificial conditions. The average number of reproduction products found in our aquaria was lower than those reported for other solitary species, used in large-scale culturing systems [5, 15]. Nevertheless, it is important to note that the current experiment used the minimum possible number of adults per aquarium, and it is probable that increasing the aquarium size and the number of adults per aquarium will result in a higher number of reproduction products.

The ability to successfully culture *P. mytiligera* in a closed system has great advantages for facilities with low or no access to the sea, or for genetic experiments in which separating the experimental animals from the natural population is of significance.

The ascidians phylogenetic position makes them a valuable model for addressing questions of chordate development and evolution [1, 6, 71]. Comparative studies have revealed that some of their developmental processes and gene signaling pathways are shared with vertebrates. For example, analysis of notochord development in *C. intestinalis* has provided valuable information on the evolution of the vertebrate form [7, 13, 71, 72]. The current study presents the first description of *P. mytiligera*’s developmental and morphological traits and provides a basis for future experimental comparative studies utilizing this species. Previous studies have revealed several modes of reproduction strategies in the genus *Polycarpa*. The species *P. cryptocarpa kroboja* found in northern Taiwan is oviparous and produces large eggs of 150–240 μm [26], while the species *P. tinctoron* from Australia is viviparous and retains its embryos in the peribranchial cavities until they are at the stage of advanced oozooids [25]. Our present findings indicate that *P. mytiligera* is oviparous, producing eggs of 200 μm in diameter. In captivity, fertilization of this species occurs during the twilight hours, and early-stage embryos can be collected at this time. Following the embryogenesis process a swimming larva hatches, featuring a typical simple body plan, similar to that in other solitary species [42]. Based on histological sections, we report that the sensory vesicle contains a single pigmented sensory, the statocyte, a unicellular organ used as a gravity receptor required for the swimming behavior [73, 74]. While in other solitary species, such as *Ciona robusta* and *Halocynthia roretzi,* a second sensory organ, the ocellus (responsible for light sensing) has been described [75–77], in *P. mytiligera* larvae only a single sense receptor was found. A single pigmented sensory organ was previously described for larvae of other *Polycarpa* species [27, 78] and for other Styelidae, such as the colonial species *Botryllus schlosseri*, in which a single organ, called a photolith, was described and is believed to be able to sense both gravity and light [79]. It remains to be established whether *Polycarpa* larvae also possess a single photolith or, rather, whether the ability to sense light has been lost in these larvae.

*P. mytiligera*’s development timeline was found to resemble that of other oviparous solitary species, such as *C. robusta* and *S. plicata* [44, 80–82]. The larvae initiate settlement and metamorphosis within about 12 h post-hatching. Three days following settlement the animal contracts its oral siphons and two protostigmata appear as it begins to feed. At this point, it has reached the juvenile stage. This life cycle coincides with that of *C. robusta,* which also opens its siphons and features the first two protostigmata 3 days post-hatching [44]; and with *Styela,* which develops into a juvenile with functional siphons 69–70 h post-hatching [80–82]. In the weeks that follow the animal increases in size and the ampules extend and spread over the substrate. Blood flow can be clearly seen in these extensions, as previously reported for the solitary ascidian *S. plicata* [83].

In their first few months of life *P. mytiligera* juveniles are transparent, enabling clear visualization of the morphological structure and developmental process that take place during these early life stages, and offering a great advantage for experimental developmental studies.

## Conclusions

With *P. mytiligera* presenting a promising model system for regenerative studies, understanding its reproduction and developmental processes and establishing appropriate culturing methods are crucial steps in rendering this species an applicable model system.

Here we provide protocols for the maintenance and spawning of adults and the rearing of larvae in an open system that utilizes fresh seawater, as well as in an artificial seawater system. *P. mytiligera* exhibits a rapid development process, with larvae initiating settlement and metamorphosis within about 12 h post-hatching. Metamorphosis into juveniles, featuring the adult body plan, was observed 3 days following settlement.

Culturing new generations in the laboratory offers the opportunity to perform controlled comparative experiments using individual animals at any stage of their life cycle, and facilitates the development of genetic and molecular tools, providing unique insights into specific developmental processes. The findings from the current study thus have important implications for future research on *P. mytiligera* as a new model for developmental and regenerative studies.

## Supplementary information

**Additional file 1: Table 1.** Sample size and environmental condition at the collection site and in system A.

**Additional file 2: Figure S1.***P. mytiligera* seasonal average number/m^2^ (±SE).

**Additional file 3: Figure S2.***P. mytiligara* gonad stracture.

**Additional file 5: Figure S3.** Histological sections of *P. mytiligera* larvae showing the connection between the nervous system components.

**Additional file 8: Figure S4.** Morphology of *P. mytiligera* juvenile stage.

## Data Availability

The datasets used and/or analyzed during the current study are available from the corresponding author upon reasonable request.
