# Supplementary material for: Spawning induction, development and culturing of the solitary ascidian Polycarpa mytiligera, an emerging model for regeneration studies
Source: Front Zool. 2020 Jun 11;17:19. doi: 10.1186/s12983-020-00365-x (PMC7288498; doi:10.1186/s12983-020-00365-x)
Supplement: Supplementary file 4 — Additional file 1: Table 1. Sample size and environmental condition at the collection site and in system A. [file 12983_2020_365_MOESM1_ESM.docx]

**Additional file 1: Table 1: sample size and environmental condition at the collection site and in system A.**

| Season | Collection site | | System A | | |
| --- | --- | --- | --- | --- | --- |
|  | Day length (hours) | Sea-water temperature (ºC) | Day length (hours) | Sea-water temperature (ºC) | Number of tested aquariums |
| Summer (August 2016, 2017) | 12:51-13:42 | 27-28 | 13 | 27 | n=4 (control) |
|  |  |  |  |  | n=5 (treatment) |
| Autumn (October 2015, 2019) | 10:13-10:54 | 24-25 | 10:30 | 24 | n=2 (control) |
|  |  |  |  |  | n=4 (treatment) |
| Winter (January 2016, 2019) | 10:05-10:37 | 21-22 | 10:15 | 21 | n=2 (control) |
|  |  |  |  |  | n=4 (treatment) |
| Spring (May 2019) | 12:30-13:24 | 22-23 | 13 | 23 | n=3 (control) |
|  |  |  |  |  | n=3 (treatment) |
