# Supplementary figures and images for: Spawning induction, development and culturing of the solitary ascidian Polycarpa mytiligera, an emerging model for regeneration studies
Source: Front Zool. 2020 Jun 11;17:19. doi: 10.1186/s12983-020-00365-x (PMC7288498; doi:10.1186/s12983-020-00365-x)

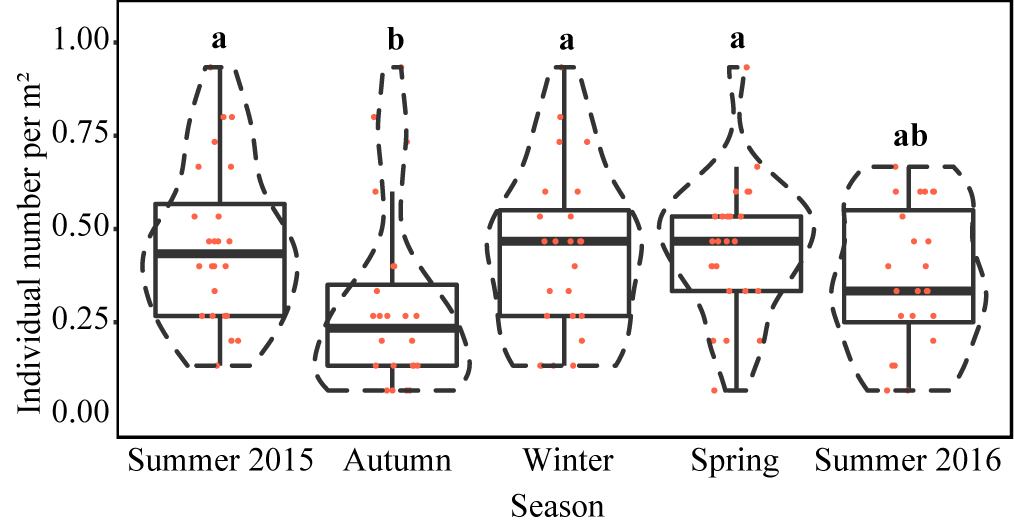

Supplement: Supplementary file 5 — Additional file 2: Figure S1.P. mytiligera seasonal average number/m2 (±SE). [file 12983_2020_365_MOESM2_ESM.tif]

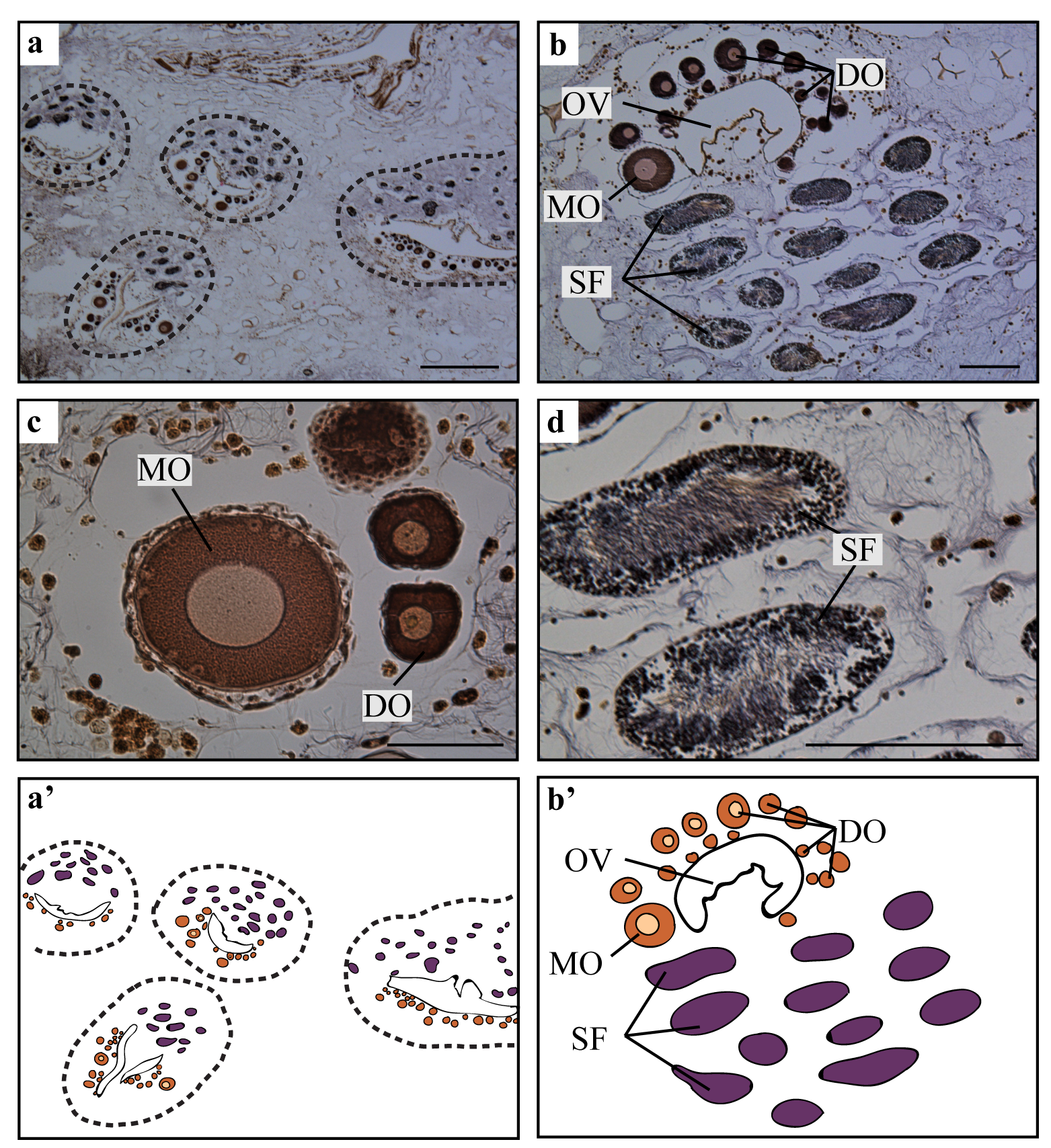

Supplement: Supplementary file 6 — Additional file 3: Figure S2.P. mytiligara gonad stracture. [file 12983_2020_365_MOESM3_ESM.tif]

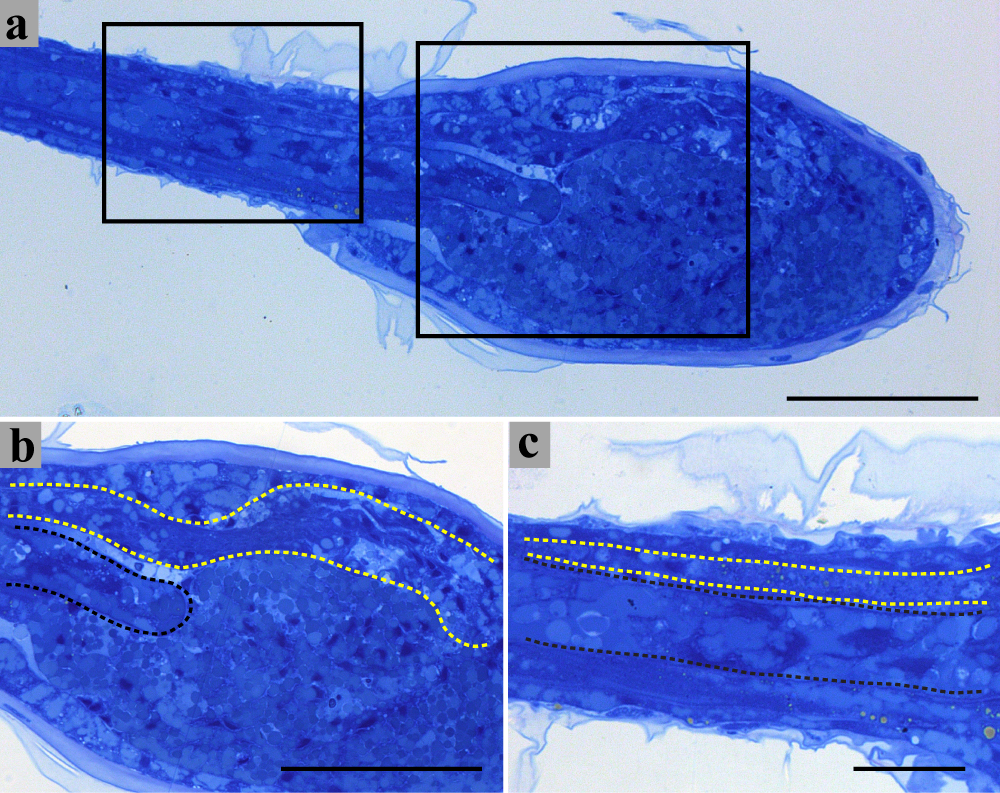

Supplement: Supplementary file 7 — Additional file 5: Figure S3. Histological sections of P. mytiligera larvae showing the connection between the nervous system components. [file 12983_2020_365_MOESM5_ESM.tif]

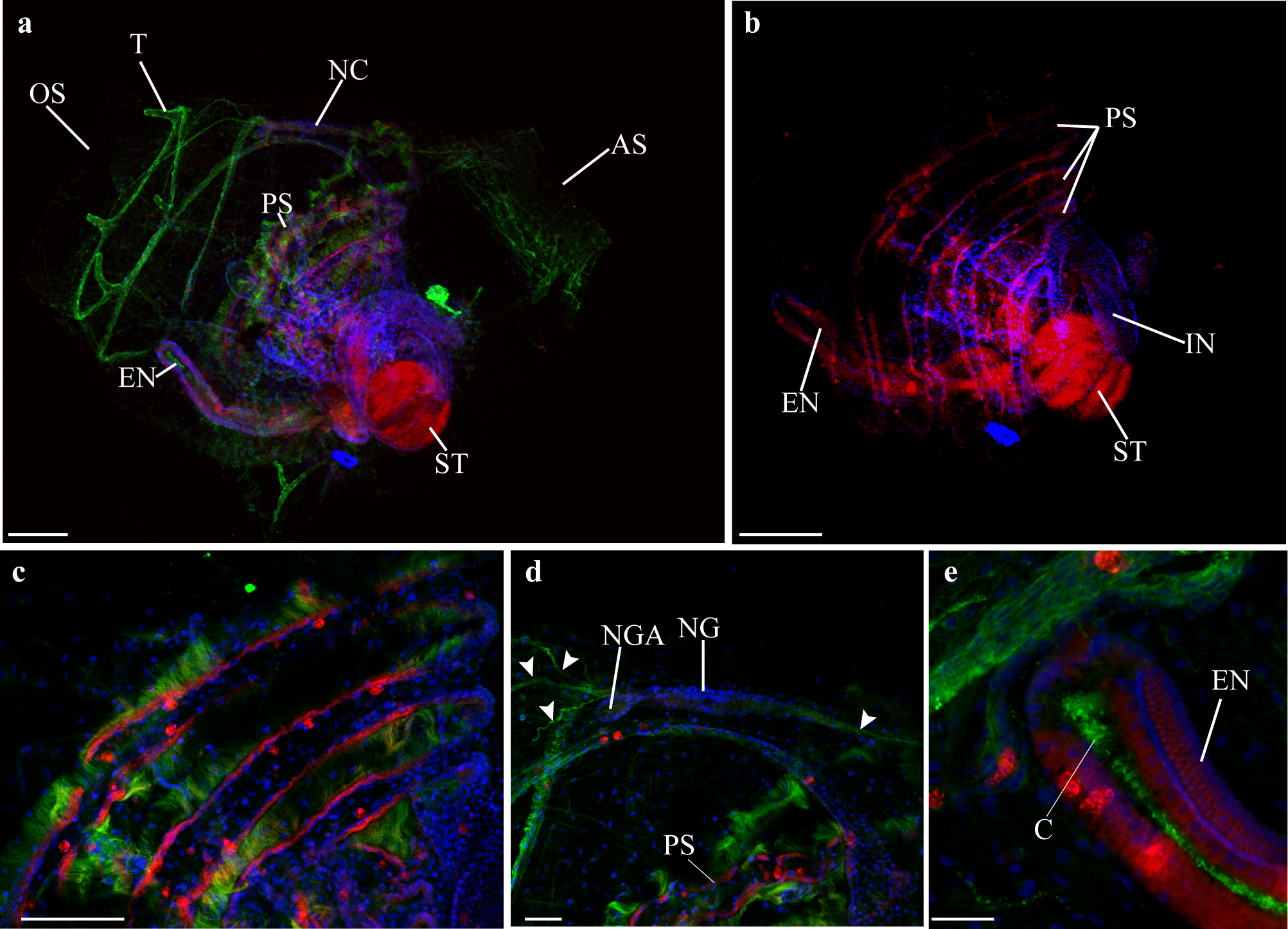

Supplement: Supplementary file 8 — Additional file 8: Figure S4. Morphology of P. mytiligera juvenile stage. [file 12983_2020_365_MOESM8_ESM.tif]
